# Impact of postoperative intravenous iron therapy on postoperative infections in older patients with severe anaemia after hip fracture surgery

**DOI:** 10.1186/s12877-023-03775-8

**Published:** 2023-02-14

**Authors:** Lene T. Hansen, Johannes Riis, Kristian H. Kragholm, Lis K. Larsen, Christian Cavallius, Marianne M. Mørch, Silas Z. Clemmensen, Maria L. Krogager, Dorte Melgaard

**Affiliations:** 1 Department of Geriatric Medicine, North Denmark Regional Hospital, Hjørring, Denmark; 2 North Denmark Regional Hospital, Hjørring, Denmark; 3 Unit of Clinical Biostatistics and Epidemiology, Aalborg University Hospital, Aalborg, Denmark; 4 Department of Cardiology, Aalborg University Hospital, Aalborg, Denmark; 5 Department of Orthopedic Surgery, Aalborg University Hospital, Hjørring, Denmark; 6 Department of Emergency Medicine, Aalborg University Hospital, Aalborg, Denmark; 7 Department of Clinical Medicine, Aalborg University, Aalborg, Denmark

**Keywords:** Postoperative anaemia, Postoperative infections, Geriatric patients, Surgery, Intravenous iron

## Abstract

**Background:**

Anaemia is common following hip fracture in ortho-geriatric patients and is associated with postoperative infections.. This study investigated whether intravenous iron supplements reduced the rate of postoperative infections within 30 days postoperatively in older adults after hip fracture surgery.

**Methods:**

This observational study included 198 ortho-geriatric patients July 2018—May 2020. In May 2019 a local guideline was implemented and recommended II therapy on the 3^rd^ postoperative day if haemoglobin concentration was < 6.5 mmol/L after hip fracture surgery.

**Results:**

The patients were divided into four treatment groups: blood transfusion (*n* = 44), IV iron (*n* = 69), blood transfusion + IV iron (*n* = 35) and no treatment (*n* = 50). The number of patients who had an infection within 30 days was similar in the two time periods (38.8% before vs. 38.9% after systematic I.V. iron supplementation, *P* = 1.00) and no significant difference according to risk of infection was found between treatment groups.

**Conclusion:**

This study documents no effect of intravenous iron supplements on postoperative infections in older adults after hip fracture surgery.

**Trial registration:**

The study was registered with the Danish Data Protection Authority (2008–58-0028) the 2^th^ of September 2019.

## Introduction

Hip fractures are common in the geriatric population with 30-day mortality estimated to be 10–15% [1, 2]. Postoperative infections after hip fracture surgery are a major factor contributing to a high mortality rate among the older patients. It has been shown that the 30-day mortality among the older patients with infection may be doubled compared to the patients without infection [3].

Bleeding from the fracture and during surgery combined with postoperative blood loss can be substantial and may lead to functional iron deficiency anaemia. Further, this may be worsened by the fact that many patients may have pre-fracture iron defiency anaemia which is an independent risk factor for bone fragility and infections [4]. A frequent treatment for postoperative anaemia is allogenic blood transfusions, however this treatment increases the risk of postoperative infections [5]. Another approach to treat postoperative anaemia and functional iron deficiency is administration of oral iron supplements or intravenous iron. Oral iron supplements are easy to administer but may have low intestinal absorption and slow bone marrow response in the older adults [6]. Intravenous iron supplements are well tolerated, and haemoglobin levels raise faster and more consistently compared to oral iron supplements and is associated with a decreased short-term mortality risk [6, 7].

Studies regarding the effects of intravenous iron supplements on postoperative infections in older patients after hip fracture surgery are few and inconsistent [8–10].

The aim of this study was to investigate whether intravenous iron supplements reduced the rate of postoperative infections in older patients undergoing hip fracture surgeries.

## Methods

This observational, single-centre study investigated the effect on postoperative infection of ferric derisomaltose (Monofer®) in ortho-geriatric patients after hip fracture surgery. The patients were included on day 3 in the post-operative course. In May 2019, a local guideline was implemented and recommended intravenous iron therapy (Monofer©) after hip fracture surgery in the Department of Orthopedic Surgery, Aalborg University Hospital, Hjørring, Denmark. The criterion for intravenous iron therapy was a haemoglobin of 6.5 mmol/L or less on the third day postoperatively; this criterion was based on a study among patients with colorectal cancer [11]. Data were collected on patients operated between July 2018 and May 2019, and on patients operated between August 2019 and May 2020. The time from May to August 2019 was dedicated to implementation of the new guideline.

We included the patients who met the following inclusion criteria; fractures of the femoral neck (ICD-10: DS720, AO: 31B1-3), pertrochanteric femoral fractures (ICD-10: DS721, AO: 31A1-3) and subtrochanteric femoral fractures (ICD-10: DS722, AO: 32A-C), age ≥ 65 years, and haemoglobin ≤ 6.5 mmol/L on third day postoperatively. Exclusion criteria were treatment with antibiotics (aside from routine prophylactic perioperative treatment) on admission or within the first three days after surgery, known serious hypersensitivity to parenteral iron products, non-iron deficiency anaemia (e.g. haemolytic anaemia), iron overload or disturbances in utilisation of iron (e.g. haemochromatosis, haemosiderosis) or decompensated liver disease.

As standard all patients received antibiotic prophylaxis; intravenous Cefuroxime (B. Braun Medical A/S) 1.5 g preoperatively and 750 mg 6, 12, and 18 h postoperatively. According to regional guidelines all patients received anticoagulant therapy and if necessary blood transfusions. To prevent infections the patients were mobilises within 24 h after surgery, urinary catheters were avoided, and screenings for dysphagia were performed [12].

The patients included after August 2019 were given intravenous iron therapy on the third postoperative day if eligible. A single dose of 20 mg/kg (max 2 g) dissolved in 100 mL sterile isotonic saline was given as an infusion lasting 30 min while the patient was observed for any side effects. Patients included before May 2019 were not given intravenous iron therapy systematically and was only administered to some patients if prescribed by a consultant in geriatric medicine.

On admission, blood samples were drawn and sent to analyses (C-reactive protein, haemoglobin, leucocytes, International Normalized Ratio, natrium, potassium, carbamide, creatinine, estimated glomerular filtration rate). On the first and third postoperative day, new blood samples were analysed (haemoglobin, natrium, potassium, carbamide, creatinine, estimated glomerular filtration rate). If needed, blood samples were sent to further analyses.

The patients’ medical records provided these demographic data: gender, age, body mass index (BMI), and habitual housing form and clinical factor scores according to the American Society of Anesthesiologists (ASA score), comorbidities classified according to Charlson’s comorbidity index (CCI), fracture type, time from admission to surgery, anaesthesia type and surgery type.

The patients were followed for 30 days from the day of surgery or until first occurrence of infection. The initiation of systemic antibiotic treatment (ATC codes starting with J01) defined the occurrence of postoperative infections. During hospitalisation, this was defined as the day of administration of the first treatment dose, and after discharge it was identified as the day that a prescription of antibiotic was first redeemed.

### Statistics

When presenting descriptive statistics, categorical variables were described using numbers and percentages and comparisons between groups was performed using Chi Squared tests. Continuous variables were checked for normality by visual inspection of histograms, normally distributed variables were reported as means and standard deviations, and groups were compared using analysis of variance.

We used G-models to determine the average risk of developing an infection across the different treatment groups and the relative risk compared to no treatment. The G-model was based on a Cox regression model with death as competing risk and standardised for distributions of age, sex, CCI, polypharmacy (more than 5 different medications), haemoglobin at the third postoperative day, type of fracture, type of surgery and anticoagulants and period of inclusion (before or after systematic I.V. iron supplementation) for all included subjects. Finally, we also performed a separate auxiliary analysis to see whether there were any differences between intra- and extracapsular types of hip fracture as they are treated differently. In this analysis we grouped patient based on whether they had osteosynthesis or arthroplasty and whether they had recieved intravenous iron or not (we did not include transfusion in this grouping as there were too few patients, but adjusted for it in the analysis instead) All analyses were performed using R version 4.0.3.

## Results

**Fig. 1:**
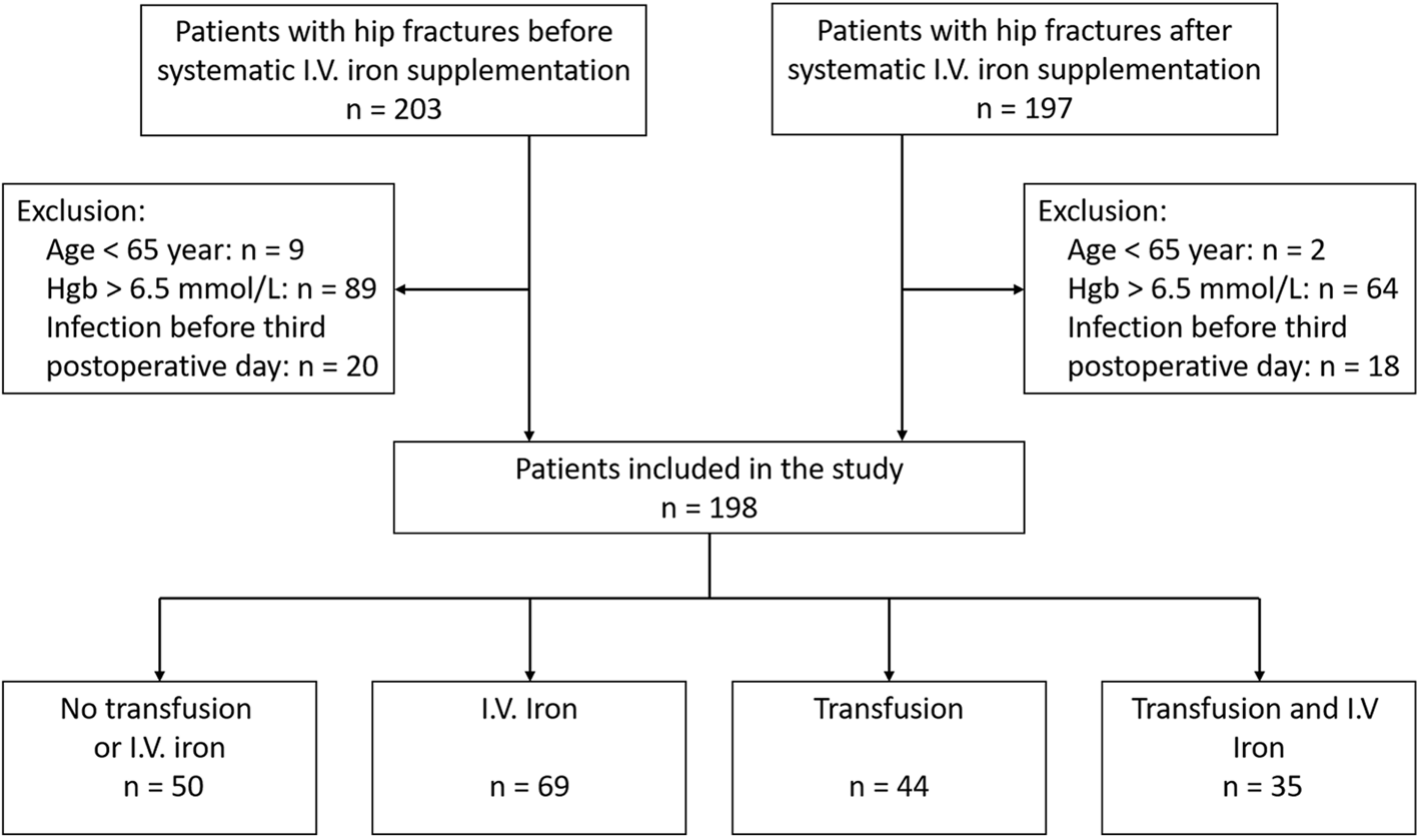
Flowchart of selection of patients for inclusion into the study

There was a total of 400 patients with hip fractures in the two periods, from which 198 were eligible to participate in the study (Fig. 1).

Following the implementation of systematic I.V. iron supplementation guidelines, most patients with haemoglobin concentrations below 6.5 mmol/L received treatment with I.V. iron supplement (90 of 113 in the present study, 79.6%). Among the 23 patients not receiving the treatment, two were discharged before administration, two refused or could not cooperate to treatment, three had contraindications for treatment, and the remaining 16 had no specified reasons.

The patients were divided into four groups based on which treatment the patients received before and on the third postoperative day. Table 1 presents the characteristics of the patients in the respective treatment groups. The number of patients who developed an infection within 30-days postoperatively (receiving systemic antibiotics) was similar in the two time periods (38.8% before and 38.9% after systematic I.V. iron supplementation, *P* > 0.99).

**Table 1:** Baseline characteristics of the study sample

	**No treatment (*n* = 50)**	**I.V. iron (*n* = 69)**	**Transfusion alone**^**a**^** (*n* = 44)**	**Both**^**a**^** (*n* = 35)**	***p*-value**
Age (mean ± SD)	82.2 ± 7.8	84.0 ± 8.8	81.6 ± 7.9	85.3 ± 8.7	0.2
Sex (female)	27 (54.0%)	37 (53.6%)	25 (56.8%)	17 (48.6%)	0.9
Fracture type					0.07
Femoral neck	25 (50.0%)	29 (42.0%)	14 (31.8%)	20 (57.1%)	
Pertrochanteric	23 (46.0%)	37 (53.6%)	19 (43.2%)	11 (31.4%)	
Subtrochanteric	2 (4.0%)	3 (4.3%)	11 (25.0%)	4 (11.4%)	
Procedure					0.12
Arthroplasty	16 (35.6%)	20 (30.3%)	16 (37.2%)	5 (14.7%)	
Gamma nail	23 (51.1%)	39 (59.1%)	26 (60.5%)	27 (79.4%)	
Dynamic hip screws	4 (8.9%)	4 (6.1%)	1 (2.3%)	1 (2.9%)	
AO screws	2 (4.4%)	3 (4.5%)	0 (0.0%)	1 (2.9%)	
Others	5 (10.0%)	3 (4.3%)	1 (2.3%)	1 (2.9%)	
BMI (mean ± SD)	24.3 ± 4.5	24.4 ± 3.9	24.6 ± 4.8	22.5 ± 3.4	0.1
*Missing*	*0*	*0*	*1*	*0*	
Charlson comorbidity index					
0	18 (36.0%)	19 (27.5%)	8 (18.2%)	8 (22.9%)	
1	10 (20.0%)	24 (34.8%)	6 (13.6%)	9 (23.4%)	
2	5 (10.0%)	11 (15.9%)	12 (27.3%)	3 (8.6%)	
≥ 3	17 (24.0%)	15 (21.7%)	18 (40.9%)	15 (42.9%)	0.03
Polypharmacy	36 (72.0%)	46 (66.7%)	40 (90.9%)	31 (88.6%)	0.006
Anticoagulant treatment	10 (20.0%)	13 (18.8%)	13 (29.5%)	11 (31.4%)	
CAS score admission (median (IQR))	6 (6, 6)	6 (6, 6)	6 (6, 6)	6 (6, 6)	0.7
*Missing*	*1*	*0*	*2*	*1*	
CAS score discharge (median (IQR))	4 (3, 6)	4 (3, 5)	3 (2, 5)	3 (2, 5)	0.4
*Missing*	*1*	*0*	*3*	*1*	
Length of stay (days) (median (IQR))	5 (4, 6)	5 (4, 6)	6 (4, 8)	6 (6, 8)	0.03
Number of transfusions^a^ (median (IQR))	0 (0, 0)	0 (0, 0)	2 (2, 3)	2 (2, 3)	< 0.001
Hgb at day 3 postoperatively (mmol/L) (median (IQR))	5.8 (5.4, 6.1)	5.8 (5.4, 6.1)	5.3 (4.7, 5.8)	5.4 (4.8, 5.9)	0.002

*Abbreviations*: *IQR* Interquartile range, *SD* Standard deviation

^a^Also includes transfusions after the 3^rd^ postoperative day

When comparing the risk of an infection within 30 days from the third postoperative day from the day of surgery in a multivariate model, there was no significant difference between treatment groups (Table 2).

**Table 2:** Risk of infection within 30 days of the third postoperative day

	**Risk (%) (95% CI)**	**Relative risk (95% CI)**	***p*-value**
**Infection within 30 days**			
No treatment	35.2 (95% CI: 19.9 – 50.5)	Reference	NA
Monofer alone	35.7 (95% CI: 21.9 – 49.4)	1.01 (95% CI: 0.37 – 1.66)	0.97
Transfusion alone	42.3 (95% CI: 26.1 – 58.5)	1.20 (95% CI: 0.54 – 1.86)	0.55
Both	46.7 (95% CI: 29.1 – 64.3)	1.33 (95% CI: 0.48 – 2.17)	0.45

Adjusted for age, sex, CCI, polypharmacy, type of fracture, type of surgery, use of anticoagulants, haemoglobin at the 3^rd^ postoperative day, and period of inclusion

In the auxiliary analysis which explored impact of operation type there were 57 patient who had arthroplasty, of whom 25 (43.9%) received intravenous iron and 139 had osteosynthesis of whom 79 (56.8%) received intravenous iron. Two had a Girdlestone type surgery and was excluded from this analysis. In patients receiving arthroplasty standardized 30-day risk infection was 41.8% (95% CI: 20% – 63%) among those receiving intravenous iron (Monofer) and 40.5% (95% CI: 21% – 61%) among those who did not (RR: 0.97, 95% CI: 0.19 – 1.74, *P* = 0.94). In patients receiving osteosynthesis standardized 30-day risk infection was 36.8% (95% CI: 26% – 48%) among those receiving intravenous iron (Monofer) and 37.5% (95% CI: 25% – 50%) among those who did not (RR: 1.02, 95% CI: 0.53 – 1.51, *P* = 0.91). As documented in Table 3 there is no significant difference in the type of antibiotics used before and after the implementation of the systematic I.V. iron supplementation guideline.

**Table 3:** Number of patients with an infection outcome within 30 days of follow-up in the two time periods

	Before systematic IV iron supplementation, *N* = 85	After systematic IV iron supplementation, *N* = 113	*P*-value
Infections	33 (38.8%)	44 (38.9%)	1.0
In hospital	18 (21.2%)	17 (15.0%)	0.4
Out of hospital	15 (17.6%)	27 (23.9%)	0.4
Type of antibiotics (% of total)	-	-	0.7
Penicillins	27 (81.8%)	33 (75.0%)	
Other Beta-lactam antibiotics	2 (6.1%)	5 (11.4%)	
Other systemic antibiotics	4 (12.1%)	6 (13.6%)	

## Discussion

Anaemia is a well-known syndrome among older patients with hip fractures and has to be diagnosed and treated comprehensively. The present study documents no effect of I.V. iron supplements vs. no intervention on post-operative infections in older patients after hip fracture surgery. There is sparse knowledge about the effect on infections of postoperative I.V. iron supplements in older patients with hip fracture. One randomized controlled trial examined perioperative I.V. iron and found a decreased blood transfusion rate but no significant reduction in postoperative infections. On the contrary, two observational studies showed a significant reduction in postoperative infections [8–10]. Similarly, a study in elective orthopaedic patients found reduced infection risk with perioperative intravenous iron [13]. The timing of I.V. iron administration (perioperative vs third postoperative day) might explain the discrepancy between these studies showing reduction in infection rates and our results showing no difference. Indeed, early intervention seemed important in a randomised trial of colorectal cancer patients who received either repeated postoperative low dose I.V. iron or a single I.V. dose in the postoperative period, since the patients with early high dose therapy had the lowest risk of infections [14].

In the present study focus is on the effect of I.V. iron supplements on post-operative infections and not a focus on the effect on bone consolidation in extracapsular hip fractures, decreased risk of falls, decreased cardiovascular risk, functional recovery or reduction of cognitive events such as delirium.

A strength of this study is the large adherence to the clinical guideline in this before and after study and that the implementation of the guideline was not presented to clinicians as a strategy to reduce infections; thus, clinical personnel were not specifically focused at reducing infections in the intervention period. A limitation of the study is that it might be under powered and follow-up might be too short to detect an effect of treatment after hospital discharge. Further, it is unknown if any of the patients had an undetected infection since the infection was defined by administered antibiotic treatment. Likewise, we cannot be sure that all patients who received antibiotics had an infection. Finally information about time to surgery, weight loading, and ongoing antiplatelets were not available.

Further, we assumed that all patients had functional iron deficiency due to bleeding, however as this study was based on clinical date, we did not have sufficient data to determine types of anaemia prior to fracture and we also had limited data on iron plasma levels, B12 vitamin and other causes of anæmia. Therefore it is possible that some patients had other types and causes of anaemia, which could have impaired the effect of empirical intravenous iron treatment and that greater effect would have been seen if these factors were systematically assessed.

## Conclusion

This study found no association between intravenous iron supplements and postoperative infections in older patients after hip fracture surgery. Further studies are needed to assess the efficacy and timing (perioperative vs. postoperative) of intravenous iron on infections in older patients after hip fracture.

## Data Availability

The dataset used and/or analysed during the present study is available from Dr. Dorte Melgaard on reasonable request.
